# Long-term supplementation of decaffeinated green tea extract does not modify body weight or abdominal obesity in a randomized trial of men at high risk for prostate cancer

**DOI:** 10.18632/oncotarget.18858

**Published:** 2017-06-29

**Authors:** Nagi B. Kumar, Roshni Patel, Julio Pow-Sang, Philippe E. Spiess, Raoul Salup, Christopher R. Williams, Michael J. Schell

**Affiliations:** ^1^ H. Lee Moffitt Cancer Center and Research Institute, Inc., MRC/CANCONT, Tampa, FL 33612, USA; ^2^ H. Lee Moffitt Cancer Center and Research Institute, Inc., WCB-GU PROG, Tampa, FL 33612, USA; ^3^ James A Haley Veterans Hospital, Tampa, FL 33612, USA; ^4^ Urologic Oncology, Research, and Robotic Surgery, University of Florida and Shands Medical Center, Jacksonville, FL 32209, USA; ^5^ H. Lee Moffitt Cancer Center and Research Institute, Inc., MRC/BIOSTAT, Tampa, FL 33612, USA

**Keywords:** green tea catechins, prostate cancer risk, obesity, body mass index and abdominal obesity

## Abstract

**Background:**

Evidence continues to demonstrate the role of obesity in prostate carcinogenesis and prognosis, underscoring the need to identify and continue to evaluate the effective interventions to reduce obesity in populations at high risk. The aim of the study was to determine the effect of daily consumption of decaffeinated green tea catechins (GTC) formulation (Polyphenon E^®^ (PolyE)) for 1 year on biomarkers of obesity in men who are at high risk for prostate cancer.

**Materials and Methods:**

A randomized, double-blinded trial was conducted targeting 97 men diagnosed with HGPIN or ASAP. Subjects were randomized to receive GTC (PolyE) (*n* = 49) or placebo (*n* = 48) for 1 year. Anthropometric data were collected at baseline, 6 and 12 months and data analyzed to observe change in weight, body mass index (indicator of obesity) and waist: hip ratio (indicator of abdominal obesity).

**Results:**

Decaffeinated GTC containing 400 mgs of the bioactive catechin, EGCG administered for 1 year to men diagnosed with ASAP and HGPIN appears to be bioavailable, well tolerated but not effective in reducing biomarkers of obesity including body weight, body mass index and waist: hip ratio.

**Conclusions:**

The results of our trial demonstrates that men who are obese and at high risk for prostate cancer should resort to effective weight management strategies to reduce obesity and not resort to ineffective measures such as taking supplements of green tea to reduce biomarkers of obesity. Changes in body mass index and abdominal obesity seen in other studies were potentially due to caffeine and not GTC.

## INTRODUCTION

It is estimated that more than one-third (34.9% or 78.6 million) of U.S. adults have a body mass index (BMI) > 30 kg/m^2^, meeting the definition of obese [1]. Although a few recent reports have continued to report conflicting data on the association between obesity and prostate cancer(PCa) risk [2–9], other well-conducted studies continue to demonstrate this association. There is evidence demonstrating that metabolic abnormality characterized by abdominal obesity has a biological rationale for increased risk of diagnosis and aggressive prostate cancer [10–21]. More recently, Folke et al. [4] reported results suggesting that obesity advances prostate carcinogenesis in men diagnosed with HGPIN. In a multi-center study reported by Cicione et al. (2016) [22], patients affected of metabolic syndrome, characterized by abdominal obesity, hypertension and insulin resistance with widespread HGPIN diagnosis were at higher risk of PCa on repeat biopsy. High-grade prostatic intraepithelial neoplasia (HGPIN) [23–26], especially mutifocal HGPIN [11–27] and atypical small acinar proliferation (ASAP) [28–30] are common histological findings on prostate biopsy, considered to be precursor lesions and predictors of subsequent prostatic adenocarcinoma. Observations have been made by other research teams, demonstrating not only a higher risk of PCa in men with precursor lesions, but worse oncologic outcomes in men with PCa, in particular with more aggressive tumor features, and biochemical recurrence [11, 27–30]. Overall, obesity, specifically abdominal obesity, is associated with increased prostate-cancer-specific morbidity and mortality. Despite the evidence linking obesity to both an increased risk of developing cancer and an increased risk of recurrence and mortality in patients with prostate cancer, to date, obesity is not considered a risk factor for prostate cancer, presence of HGPIN and/or ASAP, in addition to age, family history and race [11, 27–31]. Consequently, screening for obesity and weight management recommendations have not specifically focused on populations at high risk for prostate cancer based on established risk factors. However, the increasing evidence linking obesity to cancer risk and outcomes underscores the need for better understanding of the role of this modifiable risk factor in Prostate Cancer (PCa) etiology to optimize screening, treatment, and prevention, specifically targeting high risk populations.

Americans spend about $2 billion a year on weight-loss nutrient-derived supplements in pill form (e.g., tablets, capsules, and softgels) [32–33]. Green tea, made from the leaves of the *Camelia sinensis* species of the Theaceae family is a widely consumed beverage for centuries and is one of the most common ingredients used in supplements for weight loss [32–33]. Green tea contains a predominate form of flavonoids, polyphenolic catechins (glavan-3-ols) and includes (−)-epigallocatechin-3-gallate (EGCG), (−)-epicatechin (EC), (−)-epigallocatechin (EGC), and (−)-epicatechin-3-gallate (ECG). We have extensively reported the anti-cancer mechanism of Green tea catechins, specifically in prostate carcinogenesis [34]. In addition to catechins, green tea also contains caffeine [35–36] which may contribute to reduction in anthropometric parameters. It has been proposed that the potential mechanism by which green tea catechins may reduce body weight is by increasing energy expenditure and fat oxidation, reducing lipogenesis, and decreasing fat absorption [37–39]. However, others have observed that EGCG alone does not increase resting metabolic rate, fat oxidation, or the thermic effect of feeding [40–41]. In a recent report of overweight and obese postmenopausal population of women, one year administration of 843 mgs decaffeinated EGCG in a GTC extract, was not associated with overall reduction in obesity. However, a reduction in tissue and abdominal %fat was observed in individuals with higher BMI at baseline [42]. Thus, the current evidence regarding the efficacy of green tea catechin formulations for weight loss appears poorly understood –and limited to retrospective studies and meta-analysis of clinical trials targeting heterogeneous populations, small sample sizes, non-standardized and varying green tea catechin formulations and doses, with durations of interventions not exceeding 12–13 weeks, with the exception of the recent report in postmenopausal obese women [42].

We recently reported on the safety and effectiveness of one year administration of green tea catechins in preventing progression of early precursor lesions of PCa (HGPIN and ASAP) [43] to prostate cancer. In this study, we also demonstrated that green tea catechins administered for one year at a dose of 200 mgs EGCG BID accumulated in the plasma, reduced serum Prostate Specific Antigen (PSA), and reduced cumulative progression from HGPIN to ASAP or PCa, without producing toxicities (Kumar et al., 2015) [43]. Daily treatment with 200 mgs EGCG administered three times a day (total 600 mg/d) for one year significantly reduced progression to prostate cancer in men in the treatment arm (incidence, approximately 3%), compared to men on placebo (incidence, 30%) [44].

The rationale in this substudy [43] was to examine if GTC can, in addition to reducing progression from early precursor lesions (HGPIN and ASAP) to prostate cancer, also produce weight loss or reduction in anthropometric parameters (body weight, body mass index, waist: hip ratio), thus contributing to overall reduction in prostate cancer risk in this high risk patient population with HGPIN or ASAP.

## MATERIALS AND METHODS

### Selection and description of participants

The study and the consent procedures were approved by the institutional review boards of all participating institutions. A consort diagram depicting the number of subjects screened, enrolled, randomized and completed intervention as well as detailed materials and methods for this study have been previously reported [43]. Briefly, men between ages 30–80 with a biopsy-proven diagnosis of HGPIN and/or ASAP less than 3 months before randomization, with no history of cancer, hepatic or renal disease, restricted from taking steroid or other supplements, or more than 6–12 cups of green tea a day, were eligible. A minimum of 12 core biopsies were obtained from subjects at baseline and post intervention. All prostate biopsies were reviewed by a central pathology laboratory and all pathologists were unaware of the treatment-group assignment. Discordant interpretations were arbitrated by a referee pathologist (senior pathologist at Moffitt Cancer Center), and concordance was achieved in all cases. Participants were enrolled at the Moffitt Cancer Center, James A. Haley VA Hospital, Tampa and University of Florida, Jacksonville, Florida from September 2008 to March 2013.

### Technical information

After eligibility was confirmed and consent obtained, participants were assigned to the intervention or placebo arm (1:1 randomization) using the SRAR system, a web-delivered subject registration application, stratified by diagnosis (HGPIN or ASAP). At randomization, baseline assessments of medical history, lower urinary tract symptoms (LUTS) using the LUTS Symptoms Scale [45], quality of life (QOL), using the Rand Short-form (SF)-36 [46], serum total PSA and plasma catechin levels were obtained. Anthropometric measurements including height, weight, body mass index, waist and hip circumference were obtained, using standardized methods described previously by our group [47–49]. Body mass index (Weight kg/height in m^2^) and waist:hip ratios were calculated with the data collected.

Polyphenon E^™^ (PolyE), an investigational agent manufactured by Mitsui Norin Co., Ltd., Shizuoka, Japan, was used in this clinical trial. The active pharmaceutical ingredient of PolyE is a purified tea fraction containing 80% to 98% total catechins by weight; the main bioactive component of which is EGCG, comprising 50% to 75% of the material. PolyE contains minimal amounts of caffeine, (< 1.0%) theobromine (< 1.0%) and gallic acid (< 0.5%). The investigational product used in this study was a hard gelatin formulation containing 200 mg EGCG/capsule. PolyE and matching placebo capsules were manufactured under contract to NCI, DCP in compliance with current good manufacturing practice regulations. An investigator-initiated IND (77626 Kumar NB PI) was obtained for this agent at this dose and for this indication [43]. Subjects in both groups were provided with a standard multivitamin/mineral preparation free of charge, to assure a consistent intake of essential vitamins and minerals among all study participants during the study period. At baseline and during study participation, we obtained information on daily intake of study agents/placebo and vitamin/mineral supplements, concomitant medications. Dietary intake was obtained at baseline and monthly by conducting random weekly, 2-day 24-hour dietary recalls (gold standard for collecting dietary data) to monitor nutritional intake, including use of other green tea catechin beverages or supplement sources, to ensure compliance to study agent and dose during the study period. Food portion visuals were provided to study participants. Subjects were expected to: maintain ≥ 85% compliance with study agent intake; comply with dietary, medication and supplement restrictions; and complete the study forms and daily logs. Compliance with study agent intake was measured during monthly visits via pill counts and self-reported daily study-agent intake logs. Adherence was assessed by measuring plasma catechin levels at baseline, 6 months and end of study (EOS). A validated liquid chromatography triple quadrupole mass spectrometry (LC/MS/MS) method (Thermo Scientific, San Jose, CA) was used to determine plasma catechin levels. We were able to successfully quantitate the four catechins (EGCG, EGC, ECG and EC) using methods previously described [50–52].

Monthly assessments of nutritional and study agents intake as well as toxicity *Common Terminology Criteria for Adverse Events* (CTCAE version 4.0), concomitant medications and organ function, including hepatic panel, PT/PTT and LDH, were performed. Repeat biopsies were performed at six months for: (a) PSA velocity > 0.75 ng/ml, or; (b) documentation of a prostate nodule on digital rectal examination. All participants who did not have PCa detected on an interim biopsy underwent EOS biopsy at 1 year. Any toxicities (adverse events) occurring during the study were reviewed by the treating physician and managed according to standard medical practice. The intervention was terminated if a participant developed PCa or a serious adverse event. Blood samples, urine and tissue from diagnostic biopsy were collected for baseline measurements and banked for future studies.

Results of the primary endpoint comparing the cumulative number of PCa diagnoses at 1-year on the two study arms have been published. Additionally comparisons of overall: (a) treatment-related adverse events; (b) AEs definitely, possibly or probably related to treatment; and (c) AEs grade 3 or higher per treatment arm from baseline to 6 and 12 months have also been published [43]. The trial was registered at Clinical Trials.gov NCT00596011.

### Statistical analysis

Baseline participant characteristics were compared between the two groups using Fisher exact tests for categorical variables and Wilcoxon rank-sum tests for continuous variables. Nutritional intake data was analyzed from 2-day 24-hour dietary recalls using a 5-step multipass procedure [53–54], which has been found to assess mean energy intake within 10% of actual intake and using the frequently updated University of Minnesota Nutrition Data System-Research version (NDS-R) database for analysis of nutrient composition. Nutritional intake, plasma catechins, weight, body mass index, waist and hip circumference and wait: hip ratio, were compared by study arm from baseline to end of intervention using a 2-sided Wilcoxon rank-sum test. Trend for adverse events by group, grade and causality were compared using the Jonckheere-Terpstra test and toxicity symptoms using the Barnard unconditional test.

## RESULTS

Of a total of ninety seven (97) men enrolled, forty nine (49) subjects were randomized to the PolyE arm and 48 to the placebo arm, with 74 reaching the primary endpoint and 70 completing the 12-month intervention. Table 1 displays the baseline demographic characteristics of subjects randomized to the two intervention arms of the study [43]. Overall, subjects in the study had a mean of 2 cores positive for HGPIN or ASAP out of 12 cores sampled. There were no statistically significant differences between the two groups in demographic, clinical or behavioral risk factors, indicating that the 2 arms of the study were well matched.

**Table 1:** Demographic characteristics of all study participants randomized to the clinical trial (*N* = 97)

Variables		Poly E Number of subjects (%)	Placebo Number of subjects (%)
Race	Black Or African American	8 (40)	12 (60)
	White	41 (53)	36 (47)
Ethnicity	Hispanic	6 (67)	3 (33)
	Non-Hispanic	42 (48)	45 (52)
	Unknown	1 (100)	0 (0)
Marital Status	Divorced/Separated	5 (50)	5 (50)
	Married	37 (51)	35 (49)
	Single	7 (47)	8 (53)
Education	Bachelor Degree Or Above	20 (53)	18 (47)
	High School Or Less	12 (50)	12 (50)
	Some College/Vocational School	17 (49)	18 (51)
Employment	Employed	26 (50)	26 (50)
	Retired	14 (47)	16 (53)
	Unemployed	9 (60)	6 (40)
Age (years)	41–60	21 (62)	13 (38)
	61–75	26 (46)	30 (54)
	> 75	2 (29)	5 (71)
Baseline Diagnosis	HGPIN	32(48)	34(52)
	ASAP	17 (55)	14(45)
Family Hx of Prostate Cancer	No	42 (48)	45 (52)
	Yes	7 (70)	3 (30)
Family History of Any Cancer (First degree relative)	No	16 (49)	17 (51)
	Yes	33 (52)	31 (48)
Vitamin/Mineral supplement use	No	13 (50)	13 (50)
	Yes	36 (51)	35 (49)
Botanical biologic supplement use	No	23 (50)	23 (50)
	Yes	26 (51)	25 (49)
History of Hypertension	No	21 (47)	24 (53)
	Yes	28 (54)	24 (46)
History of Coronary Artery Disease	No	46 (51)	44 (49)
	Yes	3 (43)	4 (57)
History of Diabetes	No	41 (48)	45 (52)
	Yes	8 (73)	3 (27)

^*^Fisher's exact test for categorical variables, and Wilcoxon rank-sum test for continuous variables.

Table 2 displays comparison of other risk factors of study participants in the two study arms at baseline. Baseline characteristics did not differ between participants who completed the trial. The mean body mass index of men with HGPIN and ASAP who were randomized to both arms of study were in the overweight range (BMI = 25.0–29.9 kg/m^2^).

**Table 2:** Other Baseline Risk characteristics of all study participants (Placebo, *n* = 48; Polyphenon E, *n* = 49)

Variable	Study Arm	Median (Range)	Interquartile	Wilcoxon P^*^
On Study Age (years)	Placebo	64 (45, 78)	(60, 69.5)	0.24
	Polyphenon E	63 (45, 79)	(57, 67)	
Smoking - Pack Years	Placebo	2.5 (0, 67.5)	(0, 19.3)	0.54
	Polyphenon E	5 (0, 120)	(0, 15.)	
History of alcohol use (drinks per	Placebo	8 (0, 150)	(0, 40)	0.72
month)	Polyphenon E	12 (0, 165)	(0, 40)	
Hours of purposeful physical activity	Placebo	3.7 (0, 34)	(1.1, 7.8)	0.69
per week	Polyphenon E	3.8 (0, 24.5)	(0.9, 7.3)	
Height (cm)	Placebo	178 (151, 198)	(173, 182)	0.74
	Polyphenon E	178 (163, 193)	(175, 182)	
Weight (kg)	Placebo	92.5 (65.7, 135)	(79.4, 102)	0.96
	Polyphenon E	92.0 (59, 140)	(85.1, 99.3)	
Body Mass Index (Weight in kgs/height	Placebo	28.9 (21.4, 40.5)	(26.3, 32.3)	0.91
in M2)	Polyphenon E	29.4 (21, 41.9)	(26, 32.2)	
Waist Circumference (cm)	Placebo	103 (83, 138)	(96.3, 110)	0.87
	Polyphenon E	103 (72.6, 131)	(96.5, 110)	
Hip Circumference (cm)	Placebo	105 (90.5, 126)	(99, 110)	0.79
	Polyphenon E	105 (81.4, 128)	(97.8, 110)	
Ratio of waist to hip circumference	Placebo	1 (0.9, 1.3)	(0.9, 1)	0.69
(Waist/Hip)	Polyphenon E	1 (0.9, 1.2)	(0.9, 1)	
Serum PSA value (ng/mL)	Placebo	4.6 (0.5, 9.4)	(3.1, 6.1)	0.67
	Polyphenon E	4.5 (1.4, 9.5)	(3.5, 5.6)	

^*^Wilcoxon rank-sum *P* value.

Adherence to agent/placebo was greater than 85% as indicated by pill count, self-reported agent logs and plasma catechin concentrations. A significantly greater number of subjects in the treatment arm demonstrated increase in plasma catechin EGCG concentrations at 6 and 12 months (*p* < .0001 and *p* = .0003, respectively) (Table 3) [43]. With the exception of 2 subjects in the placebo arm, significantly higher individual change in plasma concentrations of EGCG was observed in the treatment arm at 6 and 12 months. Other catechins were non-detectable or below quantifiable levels in the plasma of all subjects.

**Table 3:** Plasma concentrations of EGCG at each time point from baseline to post intervention by study arm (*n* = 70)

	Placebo ng/ml Mean (SD)	Polyphenon E ng/ml Mean (SD)	*P* value^*^
Baseline Month 0	0	0	1.00
Month 6	0.5 (2.1)	14.7 (19.9)	< 0.0001
Month 12	1.2 (6.3)	12.3 (24.8)	0.0003

^*^P value calculated from Wilcoxon rank-sum test, 2-sided.

Table 4 provides changes in nutritional intake from baseline to post intervention in the treatment arm compared to the placebo arm. There were no statistically significant differences between groups or differences in changes from baseline to post intervention in intake of specific macro- (total calories, carbohydrates, fats and proteins) and micronutrients (vitamins, minerals) including phytonutrients.

**Table 4:** Change in Nutritional Intake of evaluable study participants by treatment group (*n* = 74)

	Baseline				End of treatment				
	Placebo *N* = 38		Poly E *N* = 36		Placebo *N* = 38		Poly E *N* = 36		
Variable	Mean	Std Dev	Mean	Std Dev	Mean	Std Dev	Mean	Std Dev	Wilcoxon p value
Energy (kcal)	1560.1	563.4	1835.0	566.2	1617.8	702.7	1855.5	712.9	0.73
Total Fat (g)	60.7	28.6	78.7	34.4	68.2	36.4	78.3	37.0	0.73
Total Carbohydrate (g)	173.9	76.4	193.7	77.3	168.8	82.7	200.5	87.1	0.64
Total Protein (g)	71.6	24.0	84.6	25.6	73.6	28.0	85.7	36.9	0.69
Animal Protein (g)	51.5	22.0	60.4	25.1	55.1	23.8	58.9	27.7	0.55
Vegetable Protein (g)	20.1	8.2	24.2	10.4	18.5	7.8	26.8	15.2	0.32
Alcohol (g)	7.3	20.2	6.0	11.5	7.9	14.3	5.2	12.4	0.43
Total Dietary Fiber (g)	13.9	9.1	17.7	8.2	13.6	6.4	18.6	10.6	1.00
Total Vitamin A Activity (IU)	4157.8	3220.2	5943.3	5254.4	3607.5	3171.8	6012.0	6160.9	0.53
Vitamin C (mg)	61.9	45.0	61.0	62.2	50.8	47.4	62.1	67.0	0.64
Vitamin D calciferol (mcg)	5.9	7.5	5.3	2.8	4.6	2.4	6.5	9.8	0.64
Vitamin E (IU)	8.3	6.1	11.1	8.2	9.2	7.3	11.1	8.0	0.91
Vitamin K (mcg)	71.9	78.8	133.0	193.9	57.4	51.7	126.5	208.2	0.60
Thiamin vitamin B1 (mg)	1.4	0.5	1.6	0.6	1.4	0.6	1.7	0.7	0.22
Riboflavin vitamin B2 (mg)	1.7	0.7	2.0	0.8	1.8	0.7	2.1	0.8	0.82
Niacin vitamin B3 (mg)	21.4	7.6	24.7	8.8	19.9	8.9	25.5	10.2	0.15
Pantothenic Acid (mg)	4.3	1.6	5.4	2.1	4.9	2.5	5.6	2.9	0.58
Vitamin B6 (mg)	2.0	1.3	2.0	0.9	1.7	0.8	2.0	0.9	0.72
Vitamin B12 (mcg)	6.2	4.5	5.4	3.0	4.4	2.1	5.3	2.6	0.23
BetaCarotene (mcg)	1447.4	1504.2	2395.8	2898.1	1223.7	1547.6	2575.2	3522.6	0.47
Alpha Carotene (mcg)	275.2	396.6	572.2	873.7	252.5	490.3	434.4	667.4	0.69
Beta Cryptoxanthin (mcg)	66.0	82.5	51.8	55.7	74.6	106.5	73.4	98.8	0.73
Lutein and Zeaxanthin (mcg)	1385.8	1844.5	2323.2	5553.7	1022.8	970.9	2596.2	6144.9	0.13
Lycopene (mcg)	2911.8	5491.2	4440.2	6589.7	4206.8	5658.2	5652.5	6461.6	0.91
Daidzein (mg)	0.5	1.7	0.3	0.5	0.6	2.2	0.3	0.5	0.34
Genistein (mg)	0.5	1.7	0.4	0.7	0.6	2.3	0.3	0.8	0.31
Caffeine (mg)	68.4	87.3	149.7	143.0	111.7	131.5	135.6	140.6	0.11
Calcium (mg)	599.6	307.5	790.9	354.7	677.4	294.8	826.7	375.2	0.45
Phosphorus (mg)	1033.6	304.4	1262.1	397.1	1078.6	415.1	1262.4	515.0	0.84
Magnesium (mg)	208.8	83.3	272.2	111.4	209.5	81.1	267.2	154.9	0.47
Iron (mg)	14.0	6.6	15.3	6.8	12.0	5.4	16.8	9.1	0.31
Zinc (mg)	10.3	6.0	12.8	5.2	10.4	4.1	12.5	5.8	0.37
Copper (mg)	0.9	0.4	1.1	0.5	0.8	0.3	1.1	0.6	0.89
Manganese (mg)	2.6	1.8	3.1	1.6	2.3	1.1	3.2	1.9	0.81
Selenium (mcg)	105.2	44.0	117.9	58.4	101.8	40.6	115.3	42.9	0.47
Sodium (mg)	2770.4	1013.3	3401.8	935.4	2913.4	1203.8	3441.4	1368.6	0.91
Potassium (mg)	1960.6	703.3	2514.3	827.9	2079.8	773.9	2504.9	1139.3	0.45

^*^P value calculated from Wilcoxon rank-sum test, 2-sided.

Table 5 displays the changes in anthropometric variables from baseline to post intervention in the treatment arm compared to the placebo arm. There were no significant changes observed with intervention on variables indicative of body mass index in the placebo arm compared to the GTC arm. Similarly, there were no reductions in waist or hip circumference nor changes in waist:hip ratio observed in the treatment arm compared to the placebo arm. No significant differences between the treatment and placebo arms were observed in toxicities, LUTS and QOL scores from baseline to end of study [43] (data not shown).

**Table 5:** Change in anthropometric variables in evaluable study participants by treatment group

		Baseline										End of Treatment							
		Placebo				Poly E						Placebo					Poly E		
Variable		N		mean	SD	N		mean		SD		N	mean		SD		N	mean	SD
Weight (kg)		37		92	14.6	36		94.77		15		34	92.35		13.3		32	93.85	15.5
Body Mass Index (kg/m2)		37		29.41	4.8	36		29.9		4.5		34	29.54		4.0		32	29.46	4.5
Waist Circumference (mm)		36		102.37	9.5	34		104.95		11.2		29	102.16		9.6		29	105	11.7
Hip Circumference (mm)		36		104.12	6.6	34		106.02		8.3		29	104.4		7.2		29	106.66	10.7
Ratio of waist to hip circumference (Waist/Hip)		36		0.98	0.1	34		0.99		0.1		28	0.98		0.1		29	0.99	0.1
Change: Post Treatment-Baseline															Wilcoxon *p* value			
Placebo								Poly E										
*N*		mean				SD		*N*		mean			SD					
33		−0.72				6.3		32		−0.11			4		0.30			
33		−0.26				2.1		32		−0.05			1.2		0.32			
29		−0.64				4.1		29		0.06			3.5		0.65			
29		0.43				3.7		29		0.14			5.4		0.45			
28		−0.01				0		29		0			0		0.45			

## DISCUSSION

To our knowledge, this is the first randomized, double-blind, placebo controlled trial, evaluating the effectiveness of a standardized decaffeinated GTC containing 400 mgs of the bioactive catechin, EGCG administered in divided doses (200 mgs EGCG BID/day) targeting men at high risk for prostate cancer, diagnosed with HGPIN and ASAP, for a duration of 1 year. Although we observed compliance to study agent and placebo, bioavailability in plasma, safety and tolerance with no indication of toxicities, decaffeinated green tea catechins at this dose and target population of overweight men at high risk for prostate cancer, failed to produce reductions in anthropometric variables, including change in weight, body mass index and abdominal obesity. Our study results are similar to trials in other heterogeneous populations that failed to observe a significant change in body mass index from use of decaffeinated green tea extracts [36]. Most recently, a one-year intervention with decaffeinated GTC in postmenopausal women failed to observe change in the body mass index.

All the studies to date that have reported reductions in body weight included interventions with caffeinated GTC. For example, in a meta-analysis of 6 randomized controlled trials with a total of 98 participants, (Hursel et al., 2013) [55] reported that caffeine, singly and in combination with catechins significantly increases energy expenditure in a dose-dependent fashion compared with placebo [38]. In a Cochrane review of 14 randomized controlled trials of green tea formulations of 1,562 overweight or obese subjects [56], with interventions ranging between 12 to 13 weeks, and doses of green tea catechins between 141 to 1,207 mg, green tea supplementation reduced body weight by a mean of 0.95 kg compared to the placebo groups. However, no significant differences were observed in subjects consuming green tea compared to placebo in the six studies reviewed which were conducted outside Japan, where study methodologies were more heterogeneous than those conducted in Japan. In another meta-analysis of 15 randomized controlled trials (6 of which examined the effects of caffeine (39–83 mg/day) with and without green tea catechins (576–690 mg/day)) on anthropometric measurements reporting a modest but significant decrease in mean body weight (1.38 kg) and waist circumference (1.93 cm) when green tea catechins were combined with caffeine over a median of 12 weeks. A similar meta-analysis of 11 randomized controlled trials of EGCG combined with caffeine for 12–13 weeks reported a mean loss of 1.31 kg body weight compared to controls [55]. Similarly, although no change in body mass index was observed, Dotal et al. [42] observed a statistically significant effect of a one year intervention with GTCs on abdominal and visceral fat. Even though statistical significance was achieved in these studies, the changes in weight as well as waist circumferences observed may not be clinically significant. Additionally, potential variations in measurement between trialists cannot be discounted [57]. Therefore, reductions in markers of abdominal fat cannot be ruled out to chance and should be interpreted with caution.

Taken together, the findings from these studies do not provide any evidence regarding the effectiveness of green tea catechins as a weight loss agent to be applicable in clinical setting [36, 56]. Despite the unclear evidence of effectiveness, the use of various formulations of green tea as a dietary supplement or as an ingredient for weight loss agents has increased significantly over the past decade. Unlike the limitations of these previous studies, there are several strengths of our current study. The strengths of our study include the randomized, double-blinded phase II clinical trial design using a standardized agent with strong preclinical evidence and early safety data, with a substantial duration of intervention for 1 year, targeting a population of men at high risk for prostate cancer, who have few options for prevention. The study was guided by an FDA IND and conducted with the same rigor by which most therapeutic agents are evaluated.

Study limitations include the evaluation of a single dose of GTC and lowest dose (200 mgs EGCG BID) tested in phase I trials to ensure safety. Additionally, the study targeted men with a relatively higher risk for prostate cancer (men with HGPIN or ASAP), limiting the generalizability of the results. A limitation of our sub study is the lack of use of accurate methods of assessing regional adiposity, which eliminates intra- and inter-examiner variation in measurements compared with manual measurements such as waist and hip circumference. Other limitations include lack of measurements of the association of GTC with steroid hormone perturbations and other metabolic markers such as insulin resistance and pro-inflammatory biomarkers that may have provided more insights into the mechanism by which GTC may decrease biomarkers of obesity.

## CONCLUSIONS

In conclusion, daily intake of a standardized, decaffeinated green tea catechin mixture containing 400 mgs EGCG (200 mgs BID) for 1 year administered to men diagnosed with ASAP and HGPIN appears to be bioavailable, well tolerated but was not statistically associated to reduction in anthropometric variables, including body weight, body mass index and waist: hip ratio. Weight reduction and body composition changes seen in other studies were potentially due to caffeine and not green tea catechins.

The growing body of evidence continues to demonstrate the association between obesity and metabolic syndrome in prostate carcinogenesis and prognosis, underscoring the need to identify and continue to evaluate effective interventions to prevent PCa progression and improve oncological outcomes in populations at high risk. Based on this evidence, screening for obesity and targeting populations at high risk for prostate cancer based on known risk factors (presence of precursor lesions, age, family history and race [11, 22–31] for interventions to reduce markers of obesity must remain a high priority. The results of our trial demonstrates that men who are obese and at high risk for prostate cancer should resort to alternate, effective weight management and physical activity strategies to reduce obesity and not resort to ineffective measures such as taking supplements of green tea to reduce biomarkers of obesity.

### Abbreviations

AE: Adverse Events; ASAP: Atypical Small Acinar Proliferation; BID: Twice (two times) a day; BMI: Body Mass Index; CTCAE: Common Terminology Criteria for Adverse Events; EC: Epicatechin; ECG: Epicatechin-3-Gallate; EGCG: Epigallocathechin-3-Gallate; EOS: End of Study; FDA IND: Federal Drug Administration Investigational New Drug (IND) Application; GTC: Green Tea C; HGPIN: High-Grade Prostatic Intgraepithelial Neoplasia; IEBs: Intermediate endpoint biomarkers; LC/MS/MS: Liquid chromatography triple quadrupole mass spectrometry; LDH: Lactate dehydrogenase; LUTS: Urinary Tract Symptoms; NDS-R: Nutrition Data System - Research; PCa; Prostate Cancer; PolyE: Polyphenon E^®^; PSA: Prostate Serum Assessment; PT/PTT: Prothrombin time and Partial Thromboplastin Time; QOL: Quality of Life; SD: Standard Deviation; SF: Short Form; SRAR: Clinical trials subject registration and randomization system.

## References

[1] Centers for Disease Control https://www.cdc.gov/obesity/data/adult.html.

[2] Augustin H (2007). Obesity and prostate cancer: an ambiguous relationship. Eur J Cancer.

[3] Baillargeon J, Rose DP (2006). Obesity, adipokines, and prostate cancer (review). Int J Oncol.

[4] Fowke JH, Motley S, Dai Q, Concepcion R, Barocas DA (2013). Association between biomarkers of obesity and risk of high-grade prostatic intraepithelial neoplasia and prostate cancer—evidence of effect modification by prostate size. Cancer Lett.

[5] Kasper JS, Giovannucci E (2006). A meta-analysis of diabetes mellitus and the risk of prostate cancer. Cancer Epidemiol Biomarkers Prev.

[6] MacInnis RJ, English DR (2006). Body size and composition and prostate cancer risk: systematic review and meta-regression analysis. Cancer Causes Control.

[7] Meyerhardt JA, Ma J, Courneya KS (2010). Energetics in colorectal and prostate cancer. J Clin Oncol.

[8] Redman MW, Tangen CM, Goodman PJ, Lucia MS, Coltman CA, Thompson IM (2008). Finasteride does not increase the risk of high-grade prostate cancer: a bias-adjusted modeling approach. Cancer Prev Res (Phila).

[9] Stewart SB, Freedland SJ (2011). Influence of obesity on the incidence and treatment of genitourinary malignancies. Urol Oncol.

[10] Bai PD, Hu MB, Xu H, Zhu WH, Hu JM, Yang T, Jiang HW, Ding Q (2015). Body mass index is associated with higher Gleason score and biochemical recurrence risk following radical prostatectomy in Chinese men: a retrospective cohort study and meta-analysis. World J Surg Oncol.

[11] Bhindi B, Locke J, Alibhai SM, Kulkarni GS, Margel DS, Hamilton RJ, Finelli A, Trachtenberg J, Zlotta AR, Toi A, Hersey KM, Evans A, van der Kwast TH, Fleshner NE (2015). Dissecting the association between metabolic syndrome and prostate cancer risk: analysis of a large clinical cohort. Eur Urol.

[12] Boehm K, Sun M, Larcher A, Blanc-Lapierre A, Schiffmann J, Graefen M, Sosa J, Saad F, Parent ME, Karakiewicz PI (2015). Waist circumference, waist-hip ratio, body mass index, and prostate cancer risk: results from the North-American case-control study Prostate Cancer & Environment Study. Urol Oncol.

[13] De Nunzio C, Simone G, Brassetti A, Mastroianni R, Collura D, Muto G, Gallucci M, Tubaro A (2016). Metabolic syndrome is associated with advanced prostate cancer in patients treated with radical retropubic prostatectomy: results from a multicentre prospective study. BMC Cancer.

[14] Gong Z, Neuhouser ML, Goodman PJ, Albanes D, Chi C, Hsing AW, Lippman SM, Platz EA, Pollak MN, Thompson IM, Kristal AR (2006). Obesity, diabetes, and risk of prostate cancer: results from the prostate cancer prevention trial. Cancer Epidemiol Biomarkers Prev.

[15] Keto CJ, Aronson WJ, Terris MK, Presti JC, Kane CJ, Amling CL, Freedland SJ (2012). Obesity is associated with castration-resistant disease and metastasis in men treated with androgen deprivation therapy after radical prostatectomy: results from the SEARCH database. BJU Int.

[16] King CR, Freedland SJ, Terris MK, Kane CJ, Amling CL, Aronson WJ, Presti JC (2007). Impact of obesity on the utility of preoperative prostate-specific antigen velocity to predict for relapse after prostatectomy: a report from the SEARCH database. Urology.

[17] Price MM, Hamilton RJ, Robertson CN, Butts MC, Freedland SJ (2008). Body mass index, prostate-specific antigen, and digital rectal examination findings among participants in a prostate cancer screening clinic. Urology.

[18] Spangler E, Zeigler-Johnson CM, Coomes M, Malkowicz SB, Wein A, Rebbeck TR (2007). Association of obesity with tumor characteristics and treatment failure of prostate cancer in African-American and European American men. J Urol.

[19] Wright ME, Chang SC, Schatzkin A, Albanes D, Kipnis V, Mouw T, Hurwitz P, Hollenbeck A, Leitzmann MF (2007). Prospective study of adiposity and weight change in relation to prostate cancer incidence and mortality. Cancer.

[20] Xiang YZ, Xiong H, Cui ZL, Jiang SB, Xia QH, Zhao Y, Li GB, Jin XB (2013). The association between metabolic syndrome and the risk of prostate cancer, high-grade prostate cancer, advanced prostate cancer, prostate cancer-specific mortality and biochemical recurrence. J Exp Clin Cancer Res.

[21] Yamoah K, Zeigler-Johnson CM, Jeffers A, Malkowicz B, Spangler E, Park JY, Whittemore A, Rebbeck TR (2016). The impact of body mass index on treatment outcomes for patients with low-intermediate risk prostate cancer. BMC Cancer.

[22] Cicione A, De Nunzio C, Tubaro A, Cantiello F, Manno S, Oliveira C, Lima E, Damiano R (2016). Metabolic syndrome diagnosis and widespread high grade prostatic intraepithelial neoplasia significantly increase prostate cancer risk: results from a multicenter biopsy study. BMC Cancer.

[23] Bostwick DG, Cheng L (2012). Precursors of prostate cancer. Histopathology.

[24] Chornokur G, Han G, Tanner R, Lin HY, Green BL, Pow-Sang J, Phelan CM (2013). High grade prostate intraepithelial neoplasia (PIN) is a PSA-independent risk factor for prostate cancer in African American men: results from a pilot study. Cancer Lett.

[25] Humphrey PA (2013). High grade prostatic intraepithelial neoplasia in prostate needle biopsy. J Urol.

[26] Lee MC, Moussa AS, Yu C, Kattan MW, Magi-Galluzzi C, Jones JS (2010). Multifocal high grade prostatic intraepithelial neoplasia is a risk factor for subsequent prostate cancer. J Urol.

[27] Gacci M, Russo GI, De Nunzio C, Sebastianelli A, Salvi M, Vignozzi L, Tubaro A, Morgia G, Serni S (2017). Meta-analysis of metabolic syndrome and prostate cancer. Prostate Cancer Prostatic Dis.

[28] Dorin RP, Wiener S, Harris CD, Wagner JR (2015). Prostate atypia: does repeat biopsy detect clinically significant prostate cancer?. Prostate.

[29] Kim TS, Ko KJ, Shin SJ, Ryoo HS, Song W, Sung HH, Han DH, Jeong BC, Seo SI, Jeon SS, Lee KS, Lee SW, Lee HM (2015). Multiple cores of high grade prostatic intraepithelial neoplasia and any core of atypia on first biopsy are significant predictor for cancer detection at a repeat biopsy. Korean J Urol.

[30] Leone A, Rotker K, Butler C, Mega A, Li J, Amin A, Schiff SF, Pareek G, Golijanin D, Renzulli JF (2015). Atypical Small Acinar Proliferation: Repeat Biopsy and Detection of High Grade Prostate Cancer. Prostate Cancer.

[31] Moyer VA, U.S. Preventive Services Task Force (2012). Screening for prostate cancer: U.S. Preventive Services Task Force recommendation statement. Ann Intern Med.

[32] Bailey RL, Gahche JJ, Miller PE, Thomas PR, Dwyer JT (2013). Why US adults use dietary supplements. JAMA Intern Med.

[33] Blanck HM, Serdula MK, Gillespie C, Galuska DA, Sharpe PA, Conway JM, Khan LK, Ainsworth BE (2007). Use of nonprescription dietary supplements for weight loss is common among Americans. J Am Diet Assoc.

[34] Connors SK, Chornokur G, Kumar NB (2012). New insights into the mechanisms of green tea catechins in the chemoprevention of prostate cancer. Nutr Cancer.

[35] Goto T, Yuko Y, Masaaki K, Hitoshi N (1996). Simultaneous analysis of individual catechins and caffeine in green tea. Journal of Chromatography.

[36] Phung OJ, Baker WL, Matthews LJ, Lanosa M, Thorne A, Coleman CI (2010). Effect of green tea catechins with or without caffeine on anthropometric measures: a systematic review and meta-analysis. Am J Clin Nutr.

[37] Grove KA, Lambert JD (2010). Laboratory, epidemiological, and human intervention studies show that tea (Camellia sinensis) may be useful in the prevention of obesity. J Nutr.

[38] Hursel R, Westerterp-Plantenga MS Catechin- and caffeine-rich teas for control of body weight in humans. Am J Clin Nutr. 2013 (Suppl).

[39] Rains TM, Agarwal S, Maki KC (2011). Antiobesity effects of green tea catechins: a mechanistic review. J Nutr Biochem.

[40] Lonac MC, Richards JC, Schweder MM, Johnson TK, Bell C (2011). Influence of short-term consumption of the caffeine-free, epigallocatechin-3-gallate supplement, Teavigo, on resting metabolism and the thermic effect of feeding. Obesity (Silver Spring).

[41] Mielgo-Ayuso J, Barrenechea L, Alcorta P, Larrarte E, Margareto J, Labayen I (2014). Effects of dietary supplementation with epigallocatechin-3-gallate on weight loss, energy homeostasis, cardiometabolic risk factors and liver function in obese women: randomised, double-blind, placebo-controlled clinical trial. Br J Nutr.

[42] Dostal AM, Arikawa A, Espejo L, Kurzer MS (2016). Long-Term Supplementation of Green Tea Extract Does Not Modify Adiposity or Bone Mineral Density in a Randomized Trial of Overweight and Obese Postmenopausal Women. J Nutr.

[43] Kumar NB, Pow-Sang J, Egan KM, Spiess PE, Dickinson S, Salup R, Helal M, McLarty J, Williams CR, Schreiber F, Parnes HL, Sebti S, Kazi A (2015). Randomized, Placebo-Controlled Trial of Green Tea Catechins for Prostate Cancer Prevention. Cancer Prev Res (Phila).

[44] Bettuzzi S, Brausi M, Rizzi F, Castagnetti G, Peracchia G, Corti A (2006). Chemoprevention of human prostate cancer by oral administration of green tea catechins in volunteers with high-grade prostate intraepithelial neoplasia: a preliminary report from a one-year proof-of-principle study. Cancer Res.

[45] Marberger M (2013). Medical management of lower urinary tract symptoms in men with benign prostatic enlargement. Adv Ther.

[46] McHorney CA, Ware JE, Raczek AE (1993). The MOS 36-Item Short-Form Health Survey (SF-36): II. Psychometric and clinical tests of validity in measuring physical and mental health constructs. Med Care.

[47] Kumar NB, Cantor A, Allen K, Riccardi D, Besterman-Dahan K, Seigne J, Helal M, Salup R, Pow-Sang J (2004). The specific role of isoflavones in reducing prostate cancer risk. Prostate.

[48] Schapira DV, Kumar NB, Lyman GH (1991). Estimate of breast cancer risk reduction with weight loss. Cancer.

[49] Schapira DV, Kumar NB, Lyman GH (1993). Variation in body fat distribution and breast cancer risk in the families of patients with breast cancer and control families. Cancer.

[50] Hung SF, Chung SD, Kuo HC (2014). Increased serum C-reactive protein level is associated with increased storage lower urinary tract symptoms in men with benign prostatic hyperplasia. PLoS One.

[51] Nam S, Smith DM, Dou QP (2001). Ester bond-containing tea polyphenols potently inhibit proteasome activity in vitro and in vivo. J Biol Chem.

[52] Wu KM, Yao J, Boring D (2011). Green tea extract-induced lethal toxicity in fasted but not in nonfasted dogs. Int J Toxicol.

[53] Jonnalagadda SS, Mitchell DC, Smiciklas-Wright H, Meaker KB, Van Heel N, Karmally W, Ershow AG, Kris-Etherton PM (2000). Accuracy of energy intake data estimated by a multiple-pass, 24-hour dietary recall technique. J Am Diet Assoc.

[54] Mitchell DC, Shacklock F (1991). Computers in nutrition: the Minnesota nutrition database. Nutr Today.

[55] Hursel R, Viechtbauer W, Westerterp-Plantenga MS (2009). The effects of green tea on weight loss and weight maintenance: a meta-analysis. Int J Obes.

[56] Jurgens TM, Whelan AM, Killian L, Doucette S, Kirk S, Foy E (2012). Green tea for weight loss and weight maintenance in overweight or obese adults. Cochrane Database Syst Rev.

[57] Nádas J, Putz Z, Kolev G, Nagy S, Jermendy G (2008). Intraobserver and interobserver variability of measuring waist circumference. Med Sci Monit.

